# Synthesis of Magnetic Ferrite Nanoparticles with High Hyperthermia Performance via a Controlled Co-Precipitation Method

**DOI:** 10.3390/nano9081176

**Published:** 2019-08-16

**Authors:** Mohamed S. A. Darwish, Hohyeon Kim, Hwangjae Lee, Chiseon Ryu, Jae Young Lee, Jungwon Yoon

**Affiliations:** 1 School of Integrated Technology, Gwangju Institute of Science and Technology, Gwangju 61005, Korea; 2 Petrochemicals Department, Egyptian Petroleum Research Institute, 1 Ahmed El-Zomor Street, El Zohour Region, Nasr City, Cairo 11727, Egypt; 3 School of Materials Science and Engineering, Gwangju Institute of Science and Technology, Gwangju 500-712, Korea; 4 Research Center for Nanorobotics in Brain, Gwangju Institute of Science and Technology, Gwangju 500-712, Korea

**Keywords:** specific loss power, magnetic ferrite, modified co-precipitation, hyperthermia

## Abstract

Magnetic nanoparticles (MNPs) that exhibit high specific loss power (SLP) at lower metal content are highly desirable for hyperthermia applications. The conventional co-precipitation process has been widely employed for the synthesis of magnetic nanoparticles. However, their hyperthermia performance is often insufficient, which is considered as the main challenge to the development of practicable cancer treatments. In particular, ferrite MNPs have unique properties, such as a strong magnetocrystalline anisotropy, high coercivity, and moderate saturation magnetization, however their hyperthermia performance needs to be further improved. In this study, cobalt ferrite (CoFe_2_O_4_) and zinc cobalt ferrite nanoparticles (ZnCoFe_2_O_4_) were prepared to achieve high SLP values by modifying the conventional co-precipitation method. Our modified method, which allows for precursor material compositions (molar ratio of Fe^+3^:Fe^+2^:Co^+2^/Zn^+2^ of 3:2:1), is a simple, environmentally friendly, and low temperature process carried out in air at a maximum temperature of 60 °C, without the need for oxidizing or coating agents. The particles produced were characterized using multiple techniques, such as X-ray diffraction (XRD), dynamic light scattering (DLS), transmission electron microscopy (TEM), ultraviolet-visible spectroscopy (UV–Vis spectroscopy), and a vibrating sample magnetometer (VSM). SLP values of the prepared nanoparticles were carefully evaluated as a function of time, magnetic field strength (30, 40, and 50 kA m^−1^), and the viscosity of the medium (water and glycerol), and compared to commercial magnetic nanoparticle materials under the same conditions. The cytotoxicity of the prepared nanoparticles by in vitro culture with NIH-3T3 fibroblasts exhibited good cytocompatibility up to 0.5 mg/mL. The safety limit of magnetic field parameters for SLP was tested. It did not exceed the 5 × 10^9^ Am^−1^ s^−1^ threshold. A saturation temperature of 45 °C could be achieved. These nanoparticles, with minimal metal content, can ideally be used for in vivo hyperthermia applications, such as cancer treatments.

## 1. Introduction

Recently, magnetic nanoparticles have shown great potential for application in various biomedical fields such as drug delivery, magnetic separation, imaging, and hyperthermia cancer treatments [1,2,3,4]. In particular, the hyperthermia capability of magnetic nanoparticles, by which they convert dissipated magnetic energy into thermal energy, enables cancer treatment. Such hyperthermia treatment depends on heating of the region affected by cancer, where the temperatures between 43 and 45 °C can be reached using magnetic nanoparticles under an alternating current (AC) magnetic field [5,6,7]. Hyperthermia can destroy the cancer cells with minimal influence on the healthy tissues, so it could potentially be used for localized, scarless, and economical treatments with few side effects. The efficacy of the hyperthermia process depends on many factors, such as properties of the magnetic nanoparticles, the magnitude of the applied AC magnetic field (*H*), and its frequency (*f*) and duration (*t*) of actuation [8,9]. The magnitude and frequency of the applied AC magnetic field must remain within safe limits to prevent unwanted side effects. A commonly prescribed safety limit is that the product of the frequency and field amplitude (*H* × *f*) should be no greater than 5 × 10^9^ Am^−1^ s^−1^ to protect the healthy tissues against excessive heating [10]. In addition, particle size is very important in determining the magnetic properties. By controlling the particle size in the transition range (about 20 nm), the particle properties can be moved from superparamagnetic to ferromagnetic, meaning that higher SLP values can be obtained [10]. Thus, it is necessary to enhance the heating ability of nanoparticles by controlling their size to obtain the required temperature rise during hyperthermia. The heating power generated per particle, the specific loss power (SLP), should be as high as possible in the injected material, and ensuring bio-safety is considered the most critical challenge to achieving desirable tumor destruction [7]. In superparamagnetic nanoparticles, the two mechanisms primarily responsible for magnetic relaxation in nanoparticles involve the physical rotation of the individual particles in the fluid (Brownian relaxation) and the collective rotation of the atomic magnetic moments within each particle (Néel relaxation) [1]. Based on these mechanisms, controlling the morphology and composition of the magnetic nanoparticles is an effective method for increasing the SLP. However, due to the problem of toxicity, the number of usable elements is severely limited [3,8]. Cobalt ferrite nanoparticles (CoFe_2_O_4_) have some advantageous unique properties, as compared to ferrous ferrite, including strong magnetocrystalline anisotropy, high coercivity (*H*c), and moderate saturation magnetization (*M*s) [4,5]. These properties, along with their high oxidative and thermal stability, make these nanoparticles attractive for hyperthermia applications. CoFe_2_O_4_ also have a variety of medical and technological applications due to their high moments at low magnetic fields, together with superparamagnetic properties [11,12]. Furthermore, they are non-toxic, biocompatible, and can be heated remotely by alternating magnetic fields. Control of their morphology and size can be achieved by varying the pH, ionic strength, coating agent, and temperature of the reaction [13,14,15,16]. Cobalt ferrite nanoparticles have been prepared by several methods, including sol-gel [11], hydrothermal [12,13], co-precipitation [14], and thermal decomposition methods [15,16]. Using the co-precipitation process, many researchers have made efforts to achieve the smallest possible particles and to improve the magnetic properties and SLP of the cobalt ferrite nanoparticles and cobalt zinc ferrite nanoparticles [14,17,18,19,20,21,22,23,24,25,26,27,28,29,30,31,32,33,34,35]. The ferromagnetic-superparamagnetic size threshold for cobalt ferrite nanoparticles has been reported by Pereira et al., who prepared superparamagnetic cobalt ferrite nanoparticles with the tuning of particle size (4.2−4.8 nm) and magnetic properties (*M*s 30.6–48.8 emu/g) using a co-precipitation method. Saturation magnetization increases with the increase in particle size until it reaches a threshold size beyond which magnetization is constant and is close to the bulk value [23]. Recently, a high SLP of 237–272 W/g_metal_ was obtained with CoFe_2_O_4_ nanoparticles via the co-precipitation method at 90 °C in the presence of chitosan as a coating agent [17]. In another report, a high SLP of 91.84 W/g_metal_ was obtained using CoFe_2_O_4_ nanoparticles via a co-precipitation method, at room temperature in the presence of a coating agent (polyethylene glycol and oleic acid) [18]. Under safe AC field conditions, a high SLP of 2131 W/g_metal_ was obtained with CoFe_2_O_4_ nanoparticles fabricated by co-precipitation (100 °C in the presence of Lauric acid) [33]. It is necessary to overcome the drawbacks of the reported synthesis processes, such as being environmentally unfavorable, expensive, dependent on high energy, and time-consuming, as well as yielding low SLP values. The solution-based synthesis method is sensitive to the composition and temperature of the precursor materials, which in turn influences the formation of the cobalt ferrite nanoparticles, particle size, and degree of crystallinity. In the current investigation, we modified the conventional co-precipitation process to make it simple, environmentally friendly, and amenable to operation under relatively low temperatures. The properties of CoFe_2_O_4_ and ZnCoFe_2_O_4_, prepared by varying the compositions of the precursor material (molar ratio of Fe^+3^:Fe^+2^:Co^+2^/Zn^+2^ of 3:2:1) at relatively low temperature (60 °C) and without oxidizing or coating agents, were investigated. The magnetic properties, optical activity, and toxicity were evaluated in an in vitro culture with NIH-3T3 fibroblasts. Ensuring the bio-safety of the nanoparticles is considered to be the greatest challenge to the development of desirable treatments. The heat generation performance of the MNPs was studied while varying the magnetic field, timing, concentration, and viscosity of the medium.

## 2. Experimental Work

### 2.1. Materials

Iron(III) chloride hexahydrate, iron(II) chloride tetrahydrate, cobalt(II) chloride hexahydrate, zinc(II) chloride, sodium citrate, and ammonium hydroxide were purchased from Sigma Aldrich (St. Louis, MO, USA). We also tested commercial iron oxides, as follows: BNF, the particles of which are magnetite with a shell of dextran dispersed in water, from micromod Partikeltechnologie GmbH (Rostock, Germany). SHA30 and SHA15, which are amine iron oxide nanoparticles dispersed in PBS with sizes of 30 and 15 nm, respectively, from Ocean Nanotech, LLC, Manufacturing Facility and R&D (San Diego, CA, USA). HyperMAG A, HyperMAG B, and HyperMAG C, which are magnetic iron oxide nanoparticles with sizes of 10.3, 11.7, and 15.2 nm, respectively, dispersed in water, from nanoTherics Ltd. (Newcastle, UK). Resovist, which are superparamagnetic iron oxide nanoparticles coated with carboxydextran, from Meito Sangyo Co., Ltd. (Nagoya, Japan).

### 2.2. Preparation of Cobalt Ferrite and Zinc Cobalt Ferrite Nanoparticles by Co-Precipitation

The synthesis process used herein is a modified version of a previously reported co-precipitation method [14,17,18,19,20,21,22,23,24,25,26,27,28,29,30,31,32,33,34,35]. The molar ratio of iron(III) chloride hexahydrate(8.1 g): iron(II) chloride tetrahydrate (3.97 g): cobalt(II) chloride hexahydrate (2.37 g) was precisely set to be 3:2:1 and mixed in 50 mL of distilled water for 15 min to obtain a homogeneous solution at room temperature. The temperature was then increased to 60 °C, and maintained for 5 min, to ensure complete homogenous mixing. With vigorous stirring, 20 ml of ammonium hydroxide (30%) was added in a dropwise manner to induce the particle growth, followed by additional stirring for 30 min at 60 °C to evaporate any excess ammonia. The black precipitate thus formed was washed several times using distilled water to remove possible ammonium salts and then dried for 24 h to obtain a cobalt ferrite nanoparticle powder.

For preparation of zinc cobalt ferrite nanoparticles, the same procedure as described above was used with the addition of zinc chloride (1.36 g).

For coating of nanoparticles with sodium citrate, 0.5 g of sodium citrate was dispersed in 25 mL water by sonication using an ultrasonic bath for 30 min to form a homogenous solution. A total of 0.1 g of nanoparticles was added to the solution and sonicated for 4 h using the ultrasonic bath.

### 2.3. Characterization

Dynamic light scattering (DLS) and zeta potential measurements were performed using a Zeta-potential and Particle Size Analyzer (ELSZ-2000; Photal Otsuka Electronics, Osaka, Japan). For DLS and zeta potential tests, suspensions of 50 mg nanoparticles in 6 mL deionized water were subjected to ultrasound (5 min) before the analyses. The magnetic properties of the samples were measured using a vibrating sample magnetometer (VSM; Lake Shore 7400 series; Lake Shore Cryotronics, Westerville, OH, USA). The X-ray diffraction (XRD) of the nanoparticles was analyzed using an X-ray diffractometer (Rigaku, Japan). The diffractometer used a copper X-ray tube and Cu Kr radiation. A scan speed of 4°/min and 2θ ranging from 5° to 65° were selected as the measurement parameters. The metal contents of the precursors were analyzed using inductively coupled plasma-optical emission spectroscopy (ICP-OES; Optima 8300, PerkinElmer, Waltham, MA, USA). A total of 0.1 g of the nanoparticles dispersed in 25 mL D.I. water was subjected to ultrasound before the analyses. The quantitation range for cost elements was 50 ppm for ICP-OES. The samples were made using an aqueous nitric acid solution. Additional dilutions were performed to make the sample concentrations according to the specified range. The morphology and structure of the materials were characterized by transmission electron microscopy (TEM) and selected area electron diffraction (SAED) (Tecnai G2S Twin; Philips, USA) at 300 keV. The optical properties were evaluated using ultraviolet-visible spectroscopy (UV–Vis spectroscopy). The clear colloid obtained after sonicating the nanoparticles dispersed in deionized water was used for the measurement and pure deionized water was used as a reference. A band gap energy (*E*_g_) is an intrinsic property of a material and it estimated from the absorption curve. Generally, electrons can jump from one band to another as long as they have the specific minimum amount of energy required for the transition. To investigate direct and indirect transitions, we plotted (αhν)^2^ against ‘hν’ and (αhν)^1/2^ against ‘hν’. The band gap energy of the material is related to the absorption coefficient ‘α’ by the Tauc relation, as follows:

(αhν)*^n^* = (absorption coefficient × energy)*^n^* = (2.303Ahν)*^n^*,

where A is a constant, hν is the photon energy, and *n* is a number (*n* = 2 and ½ for direct and indirect transitions, respectively).

Band gap energy (*E*_g_) = hc/λ,

where h is Planck’s constant (6.626 × 10^−34^ Joules/s), c is the speed of light (3.0 × 10^8^ m/s), and λ is the cut off wavelength (*E*_g_ = 1240 eV nm/λ) (energy in eV).

Heating efficiency and SLP were measured using our Lab-made system. In this setup, a function generator is used to generate a sinusoidal voltage signal which is amplified to the desired power through an AC Power amplifier, AE Techron 7224. This amplified signal is fed to a litz wire coil wound around a ferrite core to induce the alternating magnetic field (30–50 kA/m at 97 kHz). High voltage capacitors, CSM 150 capacitor, are used to set the resonant frequency and the particle temperature is measured using an Osensa optic temperature measurement cable. The SLP is calculated using the following equation; we take into account only the first few seconds, in which a quasi-adiabatic regime is assumed [29,30,31,32,33,34,35]:

SLP = (*C*_p_/*m)* × (d*T/*d*t)*,

where d*T*/d*t* is the initial gradient of the time-dependent temperature curve, *C*_p_ is the volumetric specific heat capacity of the sample solution, J/(g °C) (4.184 [water], 2.43 [glycerol]), and *m* is the mass of elements in the particles.

### 2.4. In Vitro Cytocompatibility Test

The toxicity of the prepared nanoparticles was studied by culturing NIH-3T3 cells using a WST assay [36]. The cells were seeded at a seeding density of 5 × 10^4^ cells/well in a 24-well plate and incubated for 1 day in Dulbecco’s modified Eagle’s medium (DMEM) supplemented with 10% fetal bovine serum (FBS) and 1% antibiotics. Then, the culture medium (1 mL) containing the prepared nanoparticles (0.125, 0.25, or 0.5 mg/mL) was added to the cells and incubated for an additional day. Cells without any treatment of nanoparticles were used as the control. The sample solution was removed and washed with Dulbecco’s phosphate-buffered saline (DPBS). Then, fresh culture medium (0.5 mL) and WST assay solution (0.05 mL) were added to each well and incubated for 2 h. Finally, 0.1 mL of the solution from each well was transferred to a new plate and the absorbance (*A*) at 450 nm was measured using a microplate reader. The cell viability was normalized to the control using the following formula:

Cell Viability (%) = (*A*_c_−*A*_s_)/*A*_c_ × 100,

where *A*_c_ is the absorbance of the control sample and *A*_s_ is the absorbance of the sample solution.

### 2.5. MNP Uptake (Prussian Blue Staining)

NIH-3T3 cells were seeded at a seeding density of 5 × 10^4^ cells/well in a 24-well plate and incubated for 1 day in Dulbecco’s modified Eagle’s medium (DMEM) supplemented with 10% fetal bovine serum (FBS) and 1% antibiotics. Culture media were replaced with fresh media containing 4 types of MNPs with a concentration of 12.5 µg/mL and the cells were further incubated for 24 h. Cells without any treatment of nanoparticles were used as the control. After incubation, the cells were washed twice with DPBS and fixed with 4% paraformaldehyde at room temperature for 15 min. Then, Prussian blue solution prepared with equal volumes of 4% potassium ferrocyanide (II) trihydrate and 4% HCl (in PBS) was added to each well and incubated at room temperature for 20 min. Then, the cells were washed with DPBS and imaged using a microscope.

## 3. Results and Discussion

### 3.1. Synthesis and Characterization of Magnetic Nanoparticles

**Figure 1:**
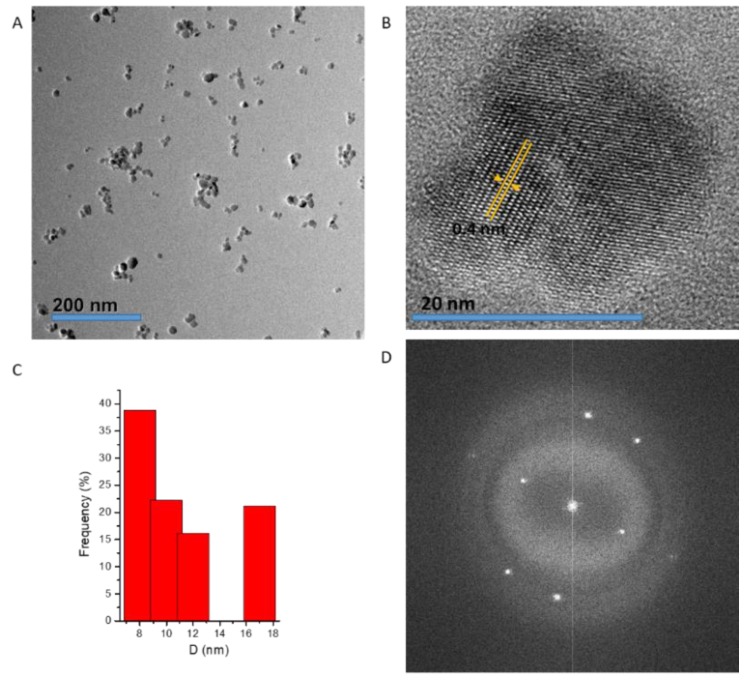

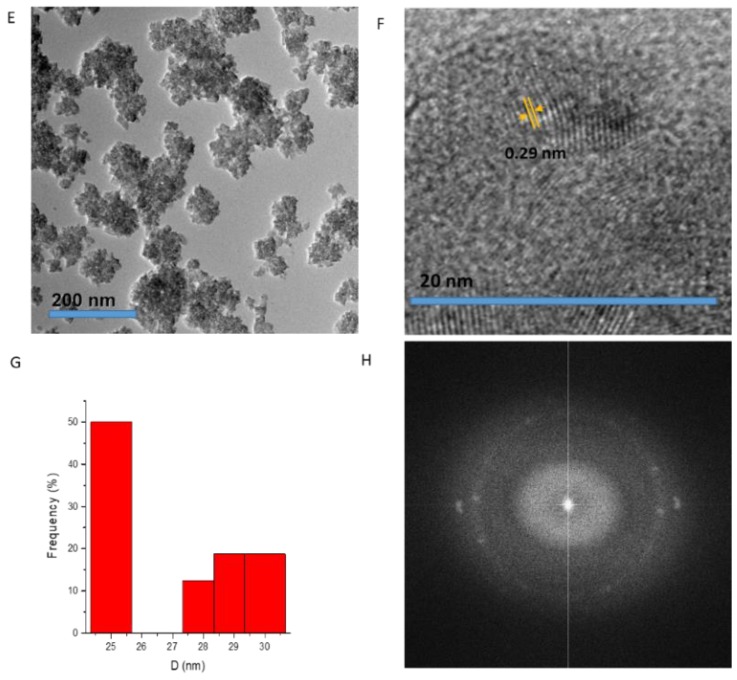
Transmission electron microscopy (TEM), selective area electron diffraction (SAED) and particle size distribution histograms for CF-MNPs (**A**–**D**) and ZCF-MNPs (**E**–**H**).

Controlling the chemical composition and size of magnetic materials is important to enhance magnetization that effectively leads to an increase in magnetic hyperthermia heating efficiency of magnetic NPs. Cobalt ferrite nanoparticles (CF-MNPs) and zinc cobalt ferrite nanoparticles (ZCF-MNPs) were prepared by a controlled co-precipitation method, which is a simple, environmentally friendly, and low-temperature method. By shaking, the prepared nanoparticles can be dispersed in water, but after a while they tend to aggregate and sediment. As shown in Figure 1, CF-MNPs had a wide size distribution with an average particle size of 8 ± 2 nm. In the case of ZCF-MNPs, the average particle size was 25 ± 5 nm, with a wide size distribution and varied agglomeration behavior. The incorporation of Zn in the NP structure lead to an increase in the particle size and agglomeration. Magnetic particles agglomerate as a result of high surface energy between the nanoparticles and magnetic dipole–dipole interactions [20,21]. The crystalline nature of the prepared nanoparticles was observed using HRTEM. The clear lattice boundary in the HRTEM image illustrates the higher crystallinity of CF-MNPs, as compared to ZCF-MNPs, which is confirmed by XRD (shown later). The corresponding selected area electron diffraction (SAED) image of nanoparticles displays the ring characteristics consistent with a structure composed of small domains with their crystallographic axes randomly oriented with respect to one another. The SAED pattern shows diffuse rings with less intensity that can be indexed to the nanoparticle plane reflections. Results indicate that our method produced smaller nanoparticles with fewer aggregations for CF-MNPs, as compared to ZCF-MNPs, due to a progressive increase in the solubility product constant of the corresponding divalent metal hydroxides [19,20,21].

**Figure 2:**
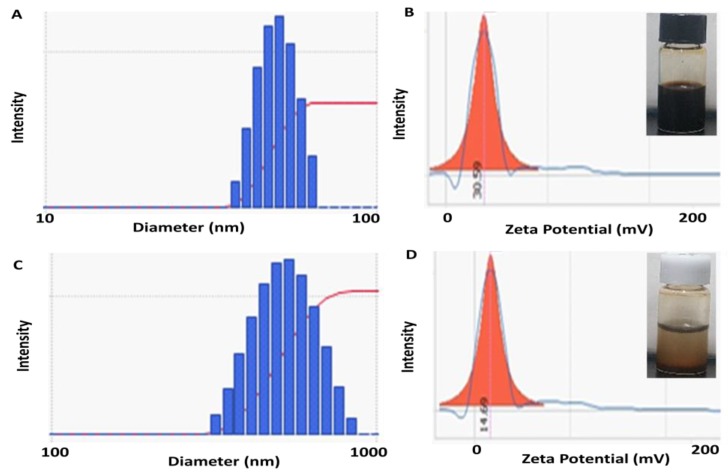
Dynamic light scattering (DLS) and zeta potential results for CF-MNPs (**A**,**B**) and ZCF-MNPs (**C**,**D**).

As shown in Figure 2, the mean hydrodynamic size obtained by DLS was 50.9 and 575 nm for CF-MNPs and ZCF-MNPs, respectively (which was higher than that obtained using TEM analysis). Wide size distribution may result from the hydrophobic nature of the prepared nanoparticles [37]. The measurements of the zeta potential were used to assess the effects of nanoparticles in the colloidal phase and their aggregates. Higher zeta potentials indicate stable nanoparticle systems [18]. The zeta potential values were +30.59 and +14.69 mV for CF-MNPs and ZCF-MNPs, respectively (Figure 2). Thus, the synthesized cobalt ferrite nanoparticles dispersed in water due to the large electrostatic repulsive forces between the particles. In contrast, zinc cobalt ferrite nanoparticles appeared less stable due to the low electrostatic repulsive forces between them. Obtaining stable colloidal systems is particularly important from the perspective of using synthesized nanoparticles in nano-medicine and biomedical applications [38].

**Figure 3:**
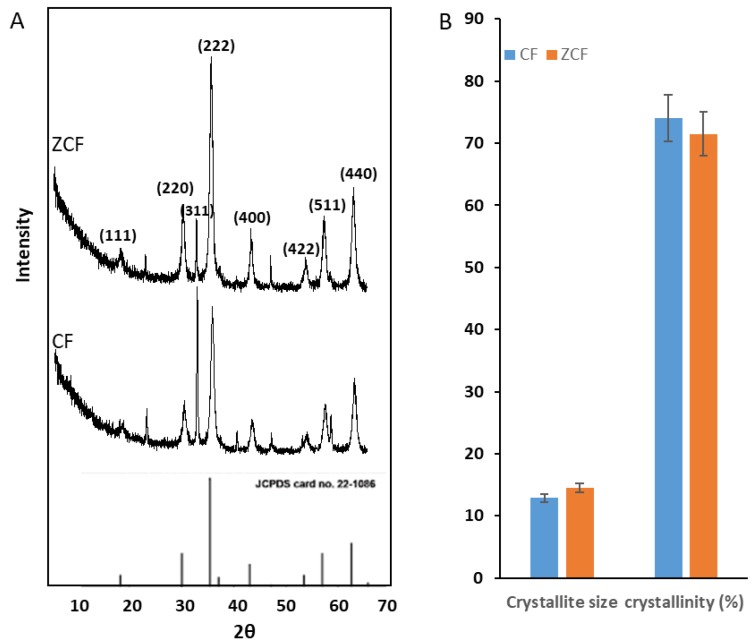
X-ray diffraction (XRD) results for CF-MNPs and ZCF-MNPs, (**A**) average crystallite size (nm) and (**B**) degree of crystallinity (%).

The crystalline properties, such as average crystallite size (nm) and degree of crystallinity, are important for the hyperthermic performance of magnetic nanoparticles. The ferrite crystalline properties of the prepared cobalt ferrite nanoparticles and zinc cobalt ferrite nanoparticles were investigated together with JCPDS data (#221086) using XRD (Figure 3). The diffraction peaks, indexed to (111), (220), (311), (222), (400), (422), (511), and (440), for the prepared nanoparticles are shown in Table 1. Shifting in peak position towards lower 2θ with decreasing intensities of peaks is caused by the presence of Zn in the structure of ZCF-MNPs.

We calculated the crystallite sizes of the nanoparticles based on the Scherrer formula [17], as follows:

Crystallite size (*D*_p_) = Kλ/(*B*cosθ),

where *D*_p_ is the average crystallite size (nm), *B* is the full width at half maximum (FWHM) of XRD peak, λ is the X-ray wavelength (1.5406 Å, K (Scherrer constant [shape parameter]): 0.89), and θ is the XRD peak position.

% Crystallinity = total area of crystalline peaks/total area of all peaks.

The crystallite sizes for the higher intensity peaks of the CF-MNPs were 29.1, 10.5, 8.7, 9.7, 8.9, and 10.4 nm, and the average crystallite size was 12.9 nm. In the case of ZCF-MNPs, the crystallite sizes of the highest intensity peaks were 31.3, 11.5, 9.7, 13.5, 10.3, and 10.8 nm, and the average crystallite size was 14.5 nm. The presence of zinc significantly affected the particle size and degree of crystallinity of the nanostructure, as shown in Figure 3. The crystallite size increased from 12.9 to 14.5 nm, while the degree of crystallinity [39] decreased from 74.03% to 71.5%, as shown in Figure 3, which is also confirmed by TEM.

Ferrites nanoparticles can be prepared from ferrous ions [25,26], ferric ions [17,18,19,20], or a mix of ferrous and ferric ions [21,31,34]. Chinnasamy et al. reported that the particle size of the ferrite powders decreased with an increase in ferric ion concentration [21]. The increase in the size of our particles caused an increase in the anisotropy energy, which in turn resulted in an increase in *H*c and *M*s.

**Figure 4:**
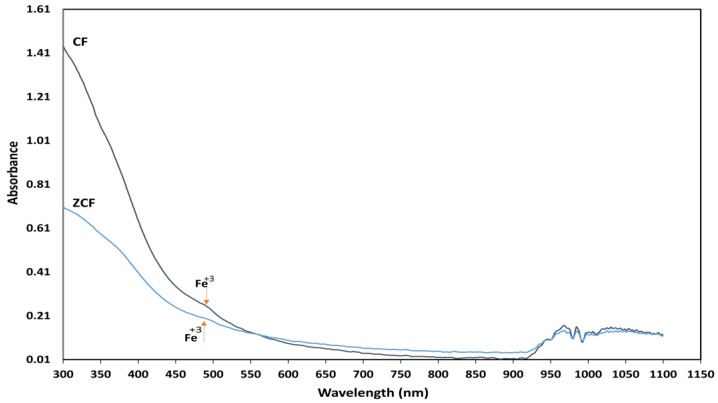
Optical absorbance spectra of CF-MNPs and ZCF-MNPs.

Photocatalysis combined with magnet heating constitutes a typical example of the so-called theranostic agents. The optical absorbance and band energy of the prepared nanoparticles were investigated by UV–Vis spectroscopy. Figure 4 shows the measured optical absorbance spectra of cobalt ferrite nanoparticles and zinc cobalt ferrite nanoparticles at ambient temperatures. The UV–visible absorption spectra of the prepared nanoparticles showed a broad absorption range (300–600 nm) in the visible wavelength range, which we attributed to d-orbital transitions of Fe^3+^. Especially, the absorption peak was close to 490 nm, which corresponds to the d-d transitions of Fe^3+^ in a tetrahedral coordination environment [40,41,42].

**Figure 5:**
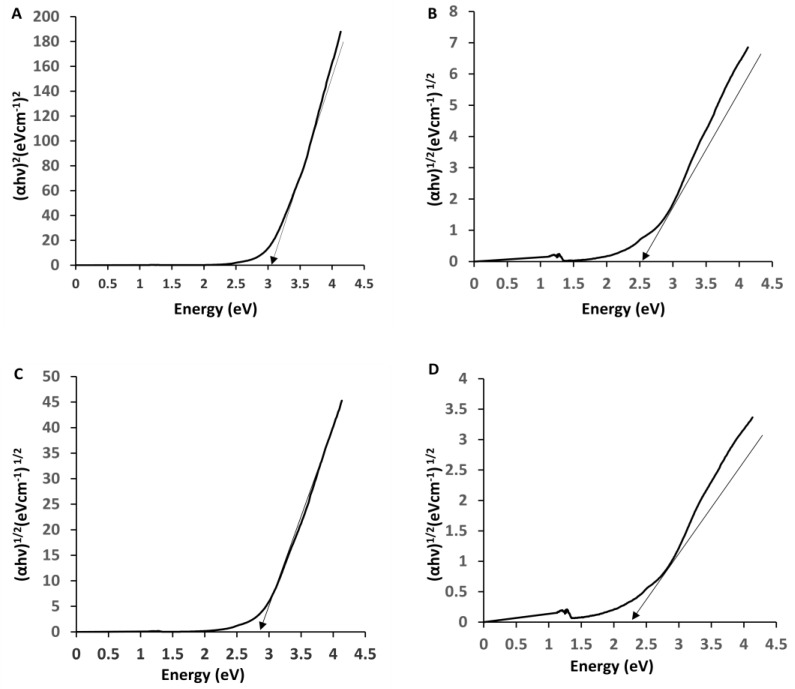
Band gap energy for CF-MNPs (direct (**A**), indirect (**B**)) and for ZCF-MNPs (direct (**C**), indirect (**D**)).

The calculated direct band gap energies of cobalt ferrite and zinc cobalt ferrite nanoparticles were 3.15 and 2.9 eV, respectively, while the respective calculated indirect band gap energies were 2.6 and 2.3 eV, as shown in Figure 5. It is clear that, as the crystallite size increased from 12.92 to 14.57 nm, the band gap energy decreased from 3.15 to 2.9 eV for cobalt ferrite and zinc cobalt ferrite nanoparticles. The band gap energy of the prepared nanoparticles varied with an inverse relationship with their sizes. This is similar to what has been reported in previous studies [43].

**Figure 6:**
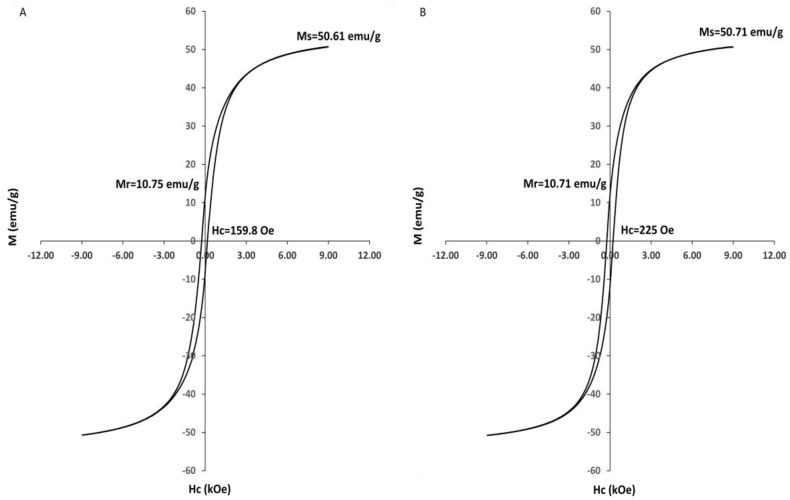
Hysteresis loops of CF-MNPs (**A**) and ZCF-MNPs (**B**).

The magnetic properties of the prepared nanoparticles were measured by VSM. The M–H results exhibit a clear hysteresis loop (Figure 6), which indicates that the magnetic nanoparticles were ferromagnetic. The cobalt ferrite nanoparticles had the following values: *M*_s_: 50.61 emu/g, *M*_r_: 10.75 emu/g and *H*_c_: 159.8 Oe. The corresponding values for the zinc cobalt ferrite nanoparticles were as follows: *M*_s_: 50.71 emu/g, *M*_r_: 10.71 emu/g, and *H*_c_: 225 Oe. *H*c and *M*s of nanoparticles increase with particle size. In addition, as in our case, the *H*c and *M*s are also increased by the shape anisotropy contribution. Thus, the increase in particle size will increase the anisotropy energy, which in turn results in an increase in the coercivity and magnetization saturation [44]. The squareness (SQ) or reduced remanence value is equal to *M*_r_/*M*_s_. When SQ is greater than or equal to 0.5, the material has a single magnetic domain structure, whereas it has a multi-domain structure when it is below 0.5. In our study, the SQ values were 0.212 and 0.211 for cobalt ferrite and zinc cobalt ferrite nanoparticles, respectively. These values less than 0.5 indicate the formation of a multi-domain structure, as has been observed previously [45].

### 3.2. Heat Generation Performance of the MNPs

**Figure 7:**
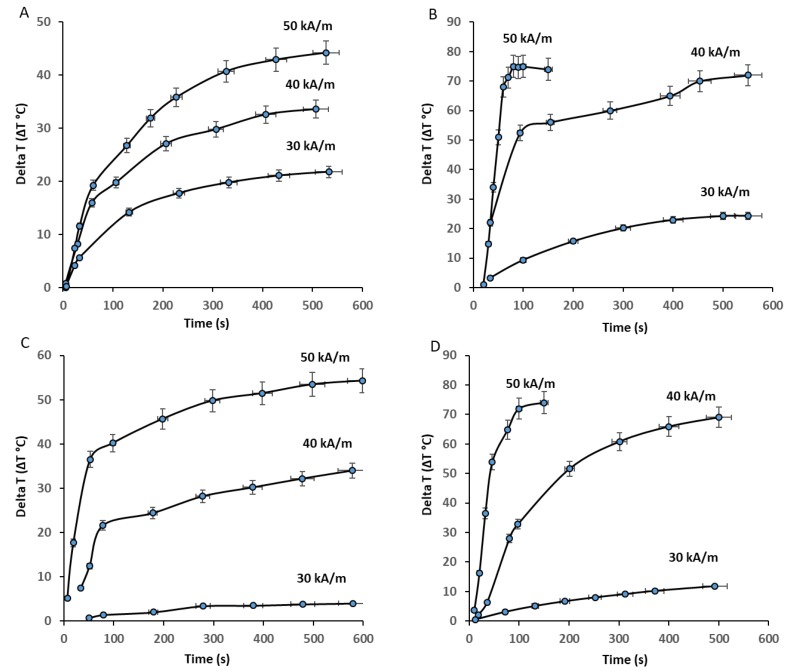
Heating profiles of CF-MNPs ((8 mg/mL) (**A**), (25 mg/mL) (**B**)) and ZCF-MNPs ((8 mg/mL) (**C**), (25 mg/mL) (**D**)) with magnetic field strengths (30, 40, or 50 kA/m) at a frequency of 97 kHz.

**Figure 8:**
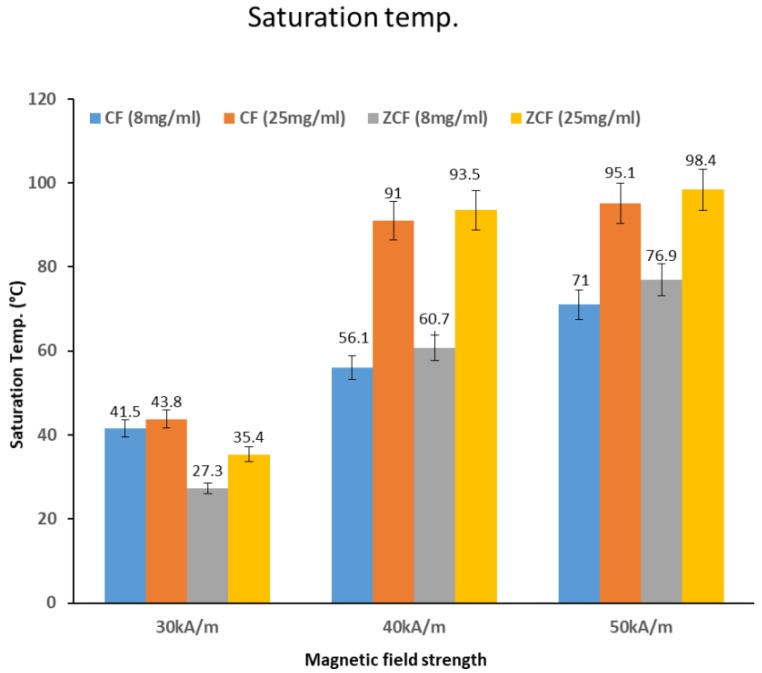
Saturation temperatures of CF-MNPs and ZCF-MNPs by concentration (8 or 25 mg/mL) and magnetic field strengths (30, 40, or 50 kA/m).

The heating properties of the prepared nanoparticles upon exposure to an AC magnetic field with frequency (97 kHz) at various magnetic field strengths (30, 40, or 50 kA/m) and concentrations (8 or 25 mg/mL) were investigated (Figure 7). The initial rise in temperature over time was approximately linear, then it slowed down gradually until saturation. The rate of heating increased with the concentration and magnetic field strength, as shown in the temperature curves in Figure 7. As the heating rate increased, the samples became heated faster according to magnetic field strength (50 > 40 > 30 kA/m) and concentration (25 > 8 mg/mL). At the highest magnetic field strength (50 kA/m), and with the higher concentration (25 mg/mL) of cobalt ferrite and zinc cobalt ferrite nanoparticles, the temperature rose to higher than 70 and 60 °C, respectively, within 1 min. At the highest magnetic field strength (50 kA/m) and lower concentration (8 mg/mL) of cobalt ferrite and zinc cobalt ferrite nanoparticles, the temperature rose higher than 45 °C within 9 and 3 min, respectively. At a moderate magnetic field strength (40 KA/m) and frequency of 97 kHz with the higher concentration (25 mg/mL) of cobalt ferrite and zinc cobalt ferrite nanoparticles, the temperature rose to over 45 °C within 2 and 3 min, respectively. At a moderate magnetic field strength (40 kA/m) and frequency of 97 kHz for both concentrations (25 and 8 mg/mL) of cobalt ferrite and zinc cobalt ferrite nanoparticles, the temperature did not reach 45 °C, even over a longer period. At the lowest magnetic field strength (30 kA/m) and frequency of 97 kHz with both concentrations (25 and 8 mg/mL) of cobalt ferrite and zinc cobalt ferrite nanoparticles, the temperature did not reach 45 °C, even over longer periods. Nanoparticles that can increase temperature to 45 °C are suitable for cancer treatment. Faster treatment with a low metal content is highly desirable for hyperthermia applications. Furthermore, for effective therapy, the temperature of cancerous tissue needs to reach 42–45 °C, while temperatures greater than 50 °C cause damage to cancer cells via thermoablation. As shown in Figure 8, the comparison of the saturated temperatures revealed that an increase in the saturation temperature of the nanoparticles was accompanied by the higher concentration and strength of the magnetic field.

The SLP is used as an indicator to measure how much energy is absorbed per mass of magnetic nanoparticles when exposed to an alternating magnetic field.

**Figure 9:**
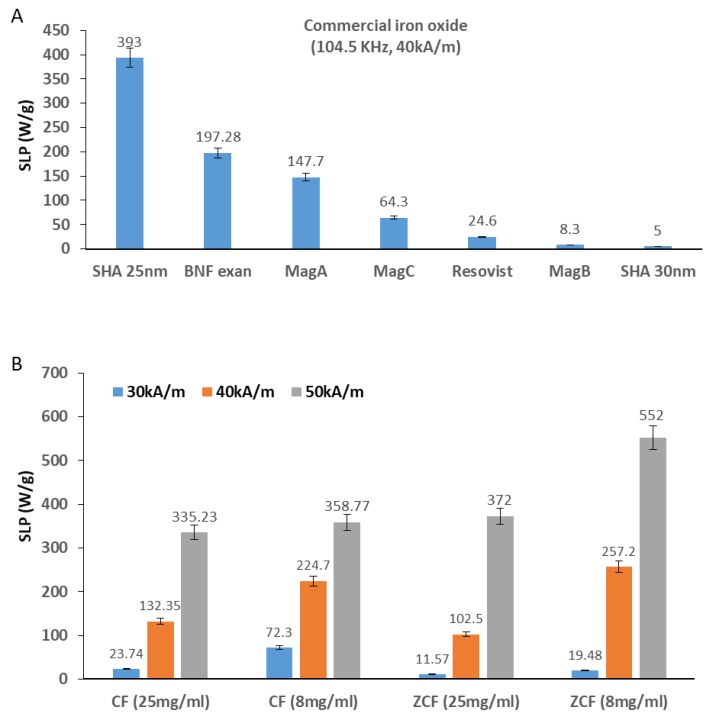
Specific loss power (SLP) values of commercial iron oxides (**A**) and cobalt ferrite nanoparticles (CF-NNPs) and zinc cobalt ferrite nanoparticles (ZCF-NNPs) (**B**).

The metal content in cobalt ferrite was Fe at 56.5% and Co at 7.81% (the total metal content was 64.31%). The metal content in zinc cobalt ferrite was Fe at 53.0%, Co at 6.31%, and Zn at 3.41% (the total metal content was 62.72%). We calculated the SLP (W/g_metals_) values based on the total metal contents. The SLP of nanoparticles in an external AC magnetic field, as mentioned earlier, can be attributed to two power loss mechanisms, as follows: Néel relaxation and Brownian relaxation. The variation in SLP values is due to several reasons, including sample size, concentrations and the magnitude and frequency of the applied field [29,30,31,32,33,34,35,45,46,47]. With respect to human exposure, it is very important to maintain the product of the magnetic field strength (*H*), and its frequency (*f*), below a threshold value. A safety limit that has been commonly prescribed is that the product of the frequency and the field amplitude should remain below *C* = *H* × *f* = 5 × 10^9^ Am^−1^s^−1^ to minimize any collateral effects of alternating magnetic fields on the human body [10]. The values of *C* in our experiments were calculated to be 2.9 × 10^9^, 3.8 × 10^9^, and 4.8 × 10^9^ Am^−1^ s^−1^ for 30, 40, and 50 kA/m, respectively. Thus, our experimental conditions for alternative magnetic field did not exceed the safety limit. In the current study, the highest SLP value obtained was 552 W/g_metal_ for zinc cobalt ferrite nanoparticles with a concentration of 8 mg/mL at 50 kA/m and 97 kHz. The lowest SLP obtained was 11.57 W/g_metal_ for zinc cobalt ferrite nanoparticles with a concentration of 25 mg/mL at 30 kA/m and 97 kHz. For ZCF, the magnetic coupling between soft and hard ferrite tune the magnetic anisotropy and, therefore, enhance the SLP values, improving the efficiency of energy conversion for hyperthermia applications. These results show the importance of changing the chemical composition of magnetic materials to enhance magnetization, which effectively leads to an increase in magnetic hyperthermia heating efficiency of magnetic NPs. SLP values are not related to concentration in this study. The observed reduction in SLP is probably caused by an extended aggregation of ferromagnetic nanoparticles, due to the application of the alternating magnetic field, and the power dissipation in the medium decreased. Thus, the heating performance was reduced. When comparing the SLP values of our nanoparticles to those of commercial iron oxide materials under the same magnetic field strength (40 kA/m), our zinc cobalt ferrite nanoparticles were found to show lower SLP values than SHA25, but higher values than the others (BNF, MagA, MagC, Resovist, MagB, and SHA30). Cobalt ferrite nanoparticles showed lower SLP values than SHA25, BNF, and MagA, but higher values than MagC, Resovist, MagB, and SHA30, as shown in Figure 9. Our results are of particular interest for heating therapy applications due to the high SLP obtained under the magnetic field parameters tested and the achievement of a saturation temperature of 45 °C. These characteristics are ideal for cancer treatment applications with minimal metal content.

**Figure 10:**
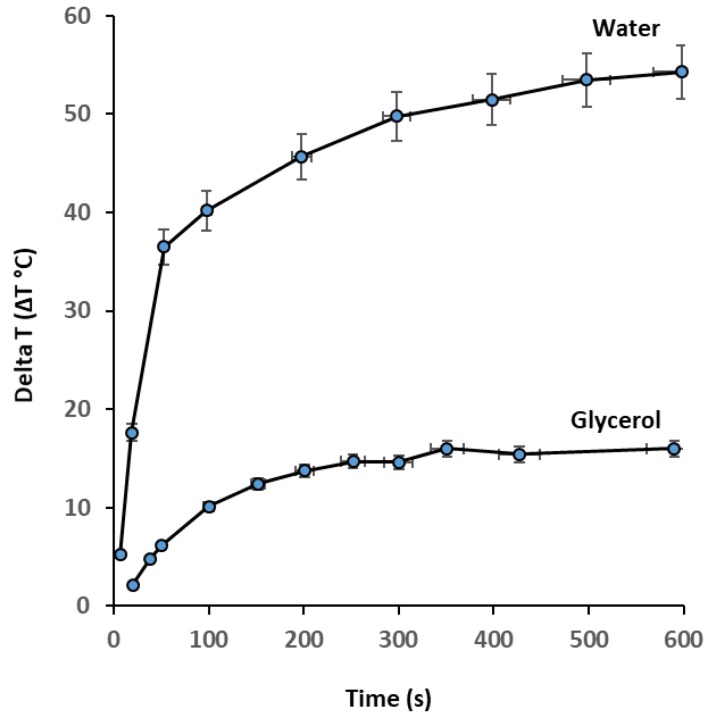
Heating profile of ZCF dispersed in water and glycerol at a concentration of 8 mg/mL, a magnetic field strength of 50 KA/m, and a frequency of 97 kHz.

It is important to differentiate between the contributions of the Néel and Brownian mechanisms to heat generation. For this purpose, we dispersed zinc cobalt ferrite nanoparticles in a high viscosity solvent. When glycerol was used as the dispersing solvent, the heating rate became very low as the exposure time increased (Figure 10). For example, in this study, the highest SLP (552 W/g_metal_) was obtained for zinc cobalt ferrite nanoparticles with a concentration of 8 mg/mL at 50 kA/m, dispersed in water. The effect of the viscosity of the solvent on the SLP value was surprising; it caused an obvious drop in SLP from 552 W/g_metal_ (water as a dispersed medium) to 35 W/g_metal_ (glycerol as a dispersed medium). Glycerol molecules contain three OH groups per molecule, which allows for extensive hydrogen bonding between the many oxygen and hydrogen atoms, which makes the substance viscous. This indicates that glycerol hinders convective heat transfer under alternating magnetic fields. The charges on the surfaces of these nanoparticles were neutralized by the surrounding solvent ions (OH^−^), providing stability. Brownian relaxation, which is due to the physical rotation of the particles within the medium, is hindered by the hydrodynamic volume and viscosity of the particles, which in turn tends to inhibit the rotation of the particles in the medium.

The Brownian relaxation time (τ_B_) is given by

τ_B_ = 3*ηV*_H_/(k_B_*T*),

where *η* is the viscosity of the fluid, *V*_H_ is the hydrodynamic volume of the particles, k_B_ is the Boltzmann constant, and *T* is the temperature [3].

The viscosity affects the heating properties via a Brownian mechanism and, thus, causes a significantly lower SLP, as reported previously [31]. This reduction is primarily due to the high viscosity of glycerol, which is 60 times that of water at room temperature [48]. It is important to note that the large amount of water molecules associated with the nanoparticles may affect their chemical stability and heat-generation ability under an AC magnetic field, which is critical for hyperthermia applications.

**Figure 11:**
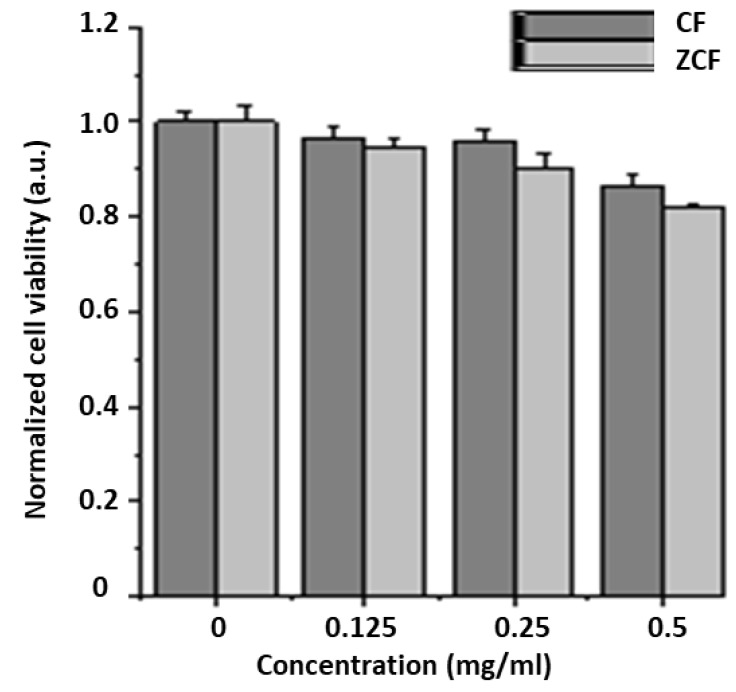
Cytocompatibility tests for CF-MNPs and ZCF-MNPs in in vitro culture with NIH-3T3.

The cytotoxicity of the cobalt ferrite and zinc cobalt ferrite nanoparticles was investigated by in vitro culture with NIH-3T3 fibroblasts (Figure 11). The cell viability remained relatively high up to concentrations of 0.5 mg/mL, for both types of nanoparticles compared to the control sample, indicating that the prepared nanoparticles have a low toxicity. The results of our cytocompatibility analysis indicate that the cell viability decreased as the concentration of zinc cobalt ferrite nanoparticles increased to 0.25 and 0.5 mg/mL. This reduction in cell viability may be attributed to the loss of colloidal stability. The incorporation of Zn in the NP structure lead to an increase in the particle size and agglomeration.

### 3.3. Characterization of Coated Magnetic Nanoparticles with Sodium Citrate

**Figure 12:**
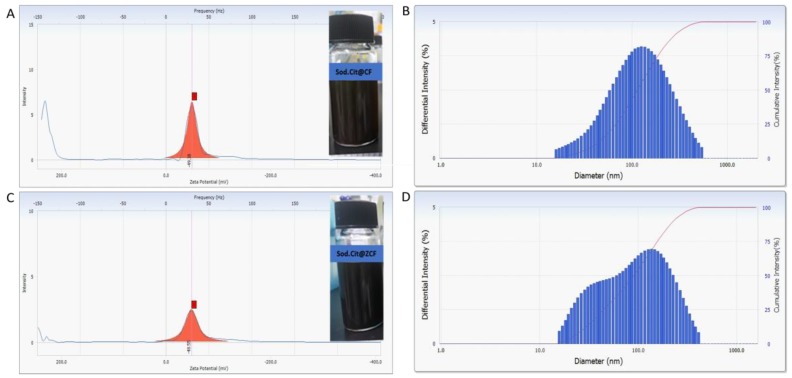
Dynamic light scattering (DLS) and zeta potential results for sodium citrate@ CF-MNPs (**A**,**B**) and sodium citrate@ ZCF-MNPs (**C**,**D**).

Modifying the chemical composition of the prepared nanoparticles by the addition of a hydrophilic surface layer (e.g., surfactant) may enhance their dispersion and heating performance. Enhancement of the heating performance of nanoparticles with size controlling and their enhanced SLP value can lead to the improvement of their effectiveness in hyperthermia. To this end, sodium citrate was selected as a surfactant for nanoparticle coating to enhance the surface charge of the MNPs to form a stable colloid with biocompatible behaviors. The effects of surfactant introduction in the colloidal phase on the possible aggregation of the nanoparticles were investigated with zeta potential measurement. The zeta potential values were −49.38 and −48.55 mV for sodium citrate@ CF-MNPs and sodium citrate@ ZCF-MNPs, respectively (Figure 12). In a neutral aqueous solution, the citrate provides negatively charged ions, which become absorbed onto the nanoparticles. The negative surface charges introduced on the surface cause repulsion among the particles, thus preventing particle aggregation and encouraging a stable dispersion. As shown in Figure 12, the mean hydrodynamic size obtained by DLS was 109.2 and 111.1 nm for sodium citrate@ CF-MNPs and sodium citrate@ ZCF-MNPs, respectively. Decrease in the amount of sodium citrate resulted in an increase in the average size of the nanoparticles. In other words, the reduction in the amount of sodium citrate decreases the citrate ions available for stabilizing the particles, which causes small particles to aggregate.

**Figure 13:**
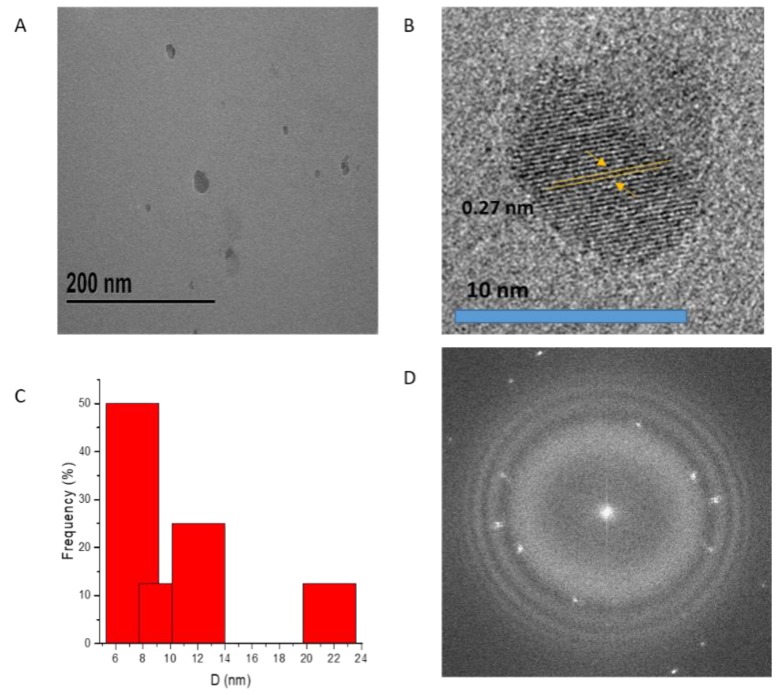

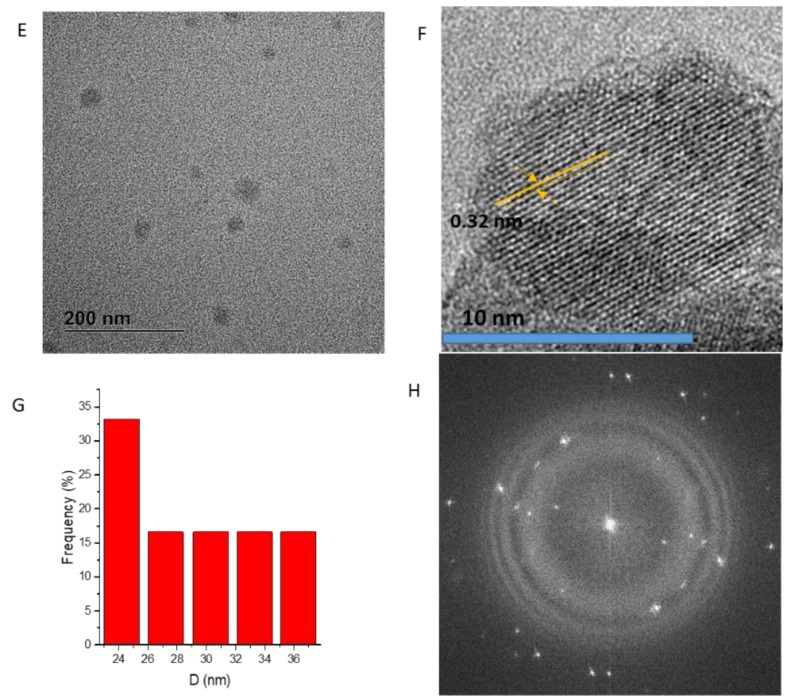
Transmission electron microscopy (TEM), selective area electron diffraction (SAED), and particle size distribution histograms for sodium citrate@ CF-MNPs (**A**–**D**) and sodium citrate@ ZCF-MNPs (**E**–**H**).

Particle size distribution and the average size of cobalt ferrite nanoparticles coated with sodium citrate (sodium citrate@ CF-MNPs) and zinc cobalt ferrite nanoparticles coated with sodium citrate (sodium citrate@ ZCF-MNPs) were investigated by TEM. As shown in Figure 13, sodium citrate decreases the aggregation between the prepared nanoparticles and the SAED pattern shows diffuse rings with high intensity that can be indexed to the nanoparticle reflections. The average particle sizes of sodium citrate@ CF-MNPs and sodium citrate@ ZCF-MNPs were 10 ± 4 nm and 30 ± 6 nm, respectively, with a wide size distribution.

**Figure 14:**
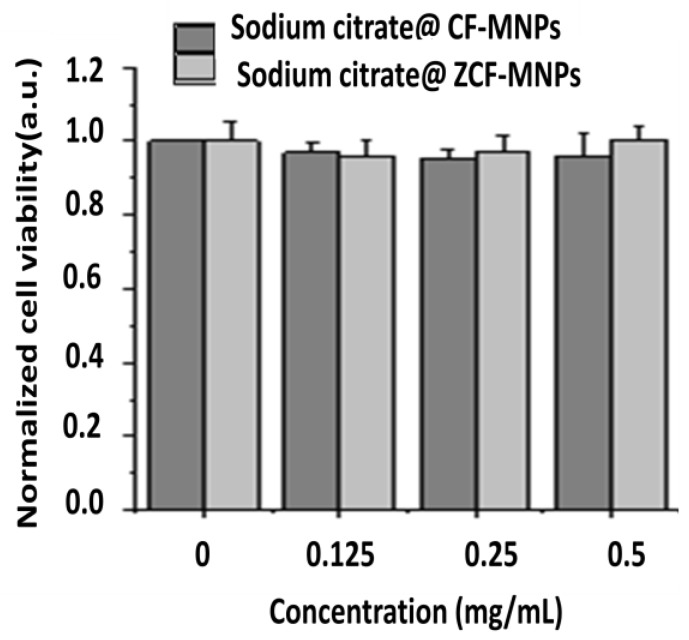
Cytocompatibility tests for sodium citrate@ CF-MNPs and sodium citrate@ ZCF-MNPs in in vitro culture with NIH-3T3.

The cytotoxicity of the coated nanoparticles was investigated by in vitro culture with NIH-3T3 fibroblasts (Figure 14). The cell viability remained relatively high up to concentrations of 0.5 mg/mL, for both types of nanoparticles compared to the control sample, indicating that the prepared nanoparticles have a low toxicity. However, these results do not prove that these nanoparticles are completely safe for in vivo applications. There may be a dissolution of the nanoparticles, which would lead to high Co^2+^ concentrations in the organism [49]. So, in-depth studies to evaluate the possible interactions between cells and our nanoparticles are currently in progress.

**Figure 15:**
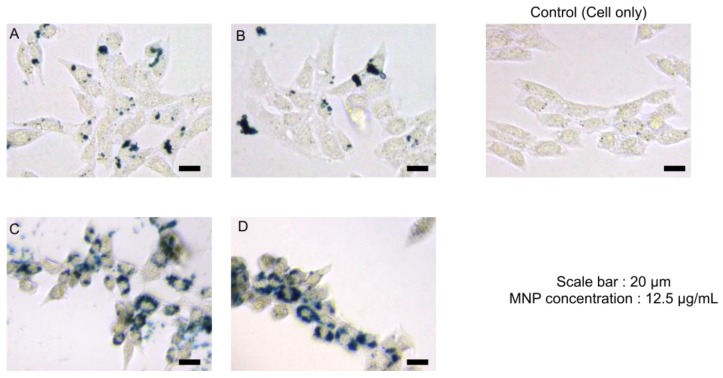
Optical micrographs of cells after the nanoparticle treatment. MNP uptake was shown by Prussian blue staining (**A**) CF-MNPs, (**B**) ZCF-MNPs (**C**) sodium citrate@ CF-MNPs, (**D**) sodium citrate@ ZCF-MNPs.

Evaluation of the interaction between cells and nanoparticles was also investigated. MNPs were stained by Prussian blue solution showing blue color (Figure 15). MNPs were located in the cell, indicating that MNPs were internalized into the cell. Citrate coated MNPs were more internalized, likely due to the lesser aggregation of MNPs.

**Figure 16:**
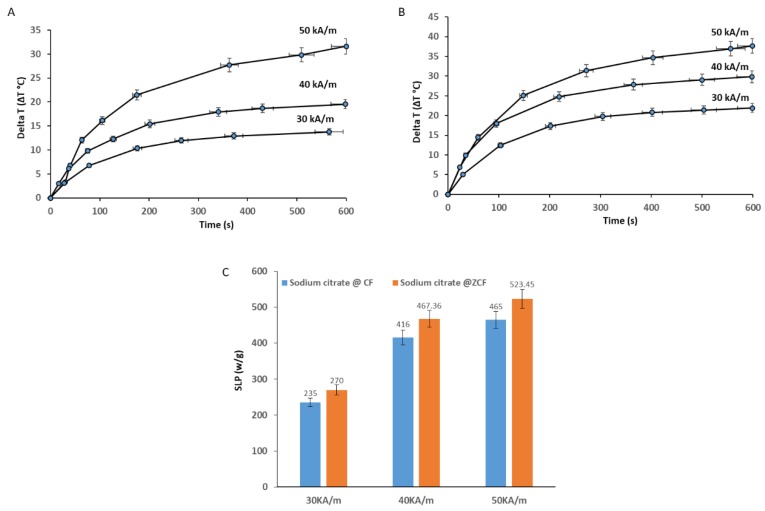
Heating profiles of sodium citrate@ CF-MNPs (**A**) and sodium citrate@ ZCF-MNPs (**B**) with magnetic field strengths (30, 40, and 50 kA/m) at a frequency of 97 kHz and SLP values (**C**).

The heating efficiency and SLP values of the prepared magnetic nanoparticles coated with sodium citrate upon exposure to an AC magnetic field with a frequency of 97 kHz at various magnetic field strengths (30, 40, and 50 kA/m) and a at concentration of 4 mg/mL were investigated (Figure 16). The total metal content in cobalt ferrite nanoparticles coated with sodium citrate (sodium citrate@ CF-MNPs) and zinc cobalt ferrite nanoparticles coated with sodium citrate (sodium citrate@ ZCF-MNPs) was 43.6% and 60.87%, respectively. The highest SLP value obtained for coated nanoparticles was 523.45 W/g_metal_ for zinc cobalt ferrite nanoparticles coated with sodium citrate with a concentration of 4 mg/mL at 50 kA/m and 97 kHz. The lowest SLP obtained for coated nanoparticles was 235 W/g_metal_ for cobalt ferrite nanoparticles coated with sodium citrate with a concentration of 4 mg/mL at 30 kA/m and 97 kHz. When comparing the SLP values of our magnetic nanoparticles coated with sodium citrate to those of commercial iron oxide materials under the same magnetic field strength (40 kA/m), our nanoparticles were found to show higher values than the commercial iron oxide materials.

We compared the results of this study to those obtained using conventional co-precipitation methods, as summarized in Table 2. The comparison was based on the synthesis conditions, magnetic properties, and the maximum SLP obtained under AC field conditions with a note on whether the safety limit was exceeded. Researchers have made many efforts to achieve the smallest nanoparticles possible at high temperatures using a coating agent and to improve the magnetic properties and SLP. Salunkhe et al. and Hoquea et al. prepared CoFe_2_O_4_ nanoparticles at room temperature via a co-precipitation method in the presence of a coating agent [18,29]. Surendra et al. prepared CoFe_2_O_4_ nanoparticles and achieved a high SLP (2131 W/g_metal_) under safe AC field conditions (18.3 kA/m, 275 kHz) [33]. In our study, the molar ratio of Fe^+3^:Fe^+2^:Co^+2^/Zn^+2^ could be simply controlled to be 3:2:1 and the maximum temperature for the synthesis could be lowered to be 60 °C in air, without the need for oxidizing or coating agents. Our nanoparticles are of particular interest for hyperthermia therapy applications due to their high SLP (552 W/g_metal_), which was achieved with minimal metal content and magnetic field conditions within the physiologically tolerable limit of 5 × 10^9^ Am^−1^ s^−1^. A temperature of 45 °C was also achieved, which is ideal for cancer treatment applications.

## 4. Conclusions

In this study, ferrite nanoparticles, namely cobalt ferrite (8 ± 2 nm) and zinc cobalt ferrite (25 ± 5 nm), were prepared using a controlled co-precipitation process. The synthesis process was a modified version of the conventional co-precipitation methods, with changes in the composition of the precursor materials (molar ratio of Fe^+3^:Fe^+2^:Co^+2^/Zn^+2^ of 3:2:1) and a simple, environmentally friendly, and low-temperature process carried out in air (the maximum temperature was 60 °C and neither oxidizing nor coating agents were required). The prepared nanoparticles exhibited optical activity with moderate magnetic saturation (50 emu/g). SLP was enhanced with respect to time, magnetic field strength, concentration, and medium viscosity. The highest SLP obtained was 552 W/g_metal_ for zinc cobalt ferrite nanoparticles at a concentration of 8 mg/mL with a magnetic field of 50 kA/m and frequency of 97 kHz, while the lowest SLP was 11.57 W/g_metal_, obtained at a concentration of 25 mg/mL with magnetic field of 30 kA/m and frequency of 97 kHz. The SLP values of our magnetic nanoparticles coated with sodium citrate were found to be higher than those for the commercial iron oxide materials. The nanoparticles exhibited high cell viability and none of the applied AC magnetic fields exceeded the physiologically tolerable limit of 5 × 10^9^ Am^−1^ s^−1^. The nanoparticles prepared using the presented method achieve high SLP and are promising for biomedical applications, such as hyperthermia cancer treatment.

## Figures and Tables

**Table 1:** The indexed diffraction peaks from XRD.

Indexed	2θ	
	CF	ZCF
(111)	18.22	18.06
(220)	30	29.86
(311)	32.46	32.40
(222)	35.42	35.22
(400)	42.96	42.88
(422)	53.52	53.32
(511)	56.96	56.78
(440)	62.58	62.51

**Table 2:** Comparison between conventional co-precipitation methods and the current study.

Sample	Temperature (°C)/Coating Agent	Size (nm)	*M*_s_ (emu/g)	SLP (W/g_metal_)	Alternating Current (AC) Field Condition (Safety Limit)	Ref.
CoFe_2_O_4_	70 (*)	14	26	–	–	[19]
CoFe_2_O_4_	90 (*)	6.5–9.7	25–42	–	–	[14]
CoFe_2_O_4_	90 (oleic acid)	14.8	52	–	–	[20]
CoFe_2_O_4_	93 ± 2 (*)	15–20	61	–	–	[21]
CoFe_2_O_4_	100 (*)	5–24	22–74	–	–	[22]
CoFe_2_O_4_	100 (*)	4.2–18.6	30–48	–	–	[23]
CoFe_2_O_4_	100 (*)	22	38	–	–	[24]
CoFe_2_O_4_	100 (*)	46–77	82–91	–	–	[25]
CoFe_2_O_4_	60 (*)	–	13	–	–	[26]
CoFe_2_O_4_	60 (*)	8	36	–	–	[27]
ZnCoFe_2_O_4_	85 (*)	6–10	14–49	–	–	[28]
CoFe_2_O_4_	90 (chitosan)	14	46.1	237–272	30 mT, 342 kHz (not safe)	[17]
CoFe_2_O_4_	Room Temp. (polyethylene glycol, oleic acid)	9.9	60.42	91.84	30 kA/m, 260 kHz (not safe)	[18]
CoFe_2_O_4_	Room Temp. (polyethylene glycol, chitosan)	7	32.3–73.1	11–289	76 mT, 400 kHz (not safe)	[29]
CoFe_2_O_4_	90 (sodium citrate)	13.56	–	82.6	9.4 kA/m, 198 kHz	[30]
CoFe_2_O_4_	80 (sodium citrate)	9.1	–	360	24.8 kA/m, 700 kHz (not safe)	[31]
CoFe_2_O_4_	90 (trisodium citrate dehydrate)	16.2	68	90.2	769 A/m, 400 kHz (safe)	[32]
CoFe_2_O_4_	100 (oleic acid) Heat treatment 100–600 °C	12–20	49–56	114–2131	18.3 kA/m, 275 kHz (safe)	[33]
CoFe_2_O_4_	90–95 (lauric acid)	9–10	59.56	51.8	15 kA/m, 300 kHz (safe)	[34]
ZnCoFe_2_O_4_	80 (*)	13	70.23	114.98	335.2 Oe, 265 kHz (not safe)	[35]
CoFe_2_O_4_	60 (*)	8	50.61	358.77	50 kA/m, 97 kHz (safe)	Current study
ZnCoFe_2_O_4_	60 (*)	25	50.71	552	50 kA/m, 97 kHz (safe)	Current study
CoFe_2_O_4_	60 (sodium citrate)	10	–	465	50 kA/m, 97 kHz (safe)	Current study
ZnCoFe_2_O_4_	60 (sodium citrate)	30	–	523.45	50 kA/m, 97 kHz (safe)	Current study

*: without coating agent; –: not measured.
